# Analysis of contact resistance in single-walled carbon nanotube channel and graphene electrodes in a thin film transistor

**DOI:** 10.1186/s40580-017-0130-1

**Published:** 2017-12-20

**Authors:** Jinwook Baek, Travis G. Novak, Houngkyung Kim, Jinsup Lee, Byoungwook Jang, Junseok Lee, Seokwoo Jeon

**Affiliations:** Department of Materials Science and Engineering, Korea Advanced Institute of Science and Technology (KAIST), 335 Gwahangno, Yuseong-gu, Daejeon, 305-701 Republic of Korea

## Abstract

In this work, we present the experimental investigation on the contact resistance of graphene/single-walled carbon nanotube (SWCNT) junction using transfer length method with the simple equivalent circuit model. We find that p–n like junctions are formed in graphene/SWCNT transistors, and the contact resistance in the junction is observed to be ~ 494 and ~ 617 kΩ in case of metallic SWCNT (m-SWCNT) and semiconducting SWCNT (s-SWCNT), respectively. In addition, the contact resistance increases from 617 to 2316 kΩ as V_g_ increases from − 30 to − 10 V. Through our study, high carrier density induced from doping in both graphene and SWCNT leads to low contact resistance. This development of contact engineering, namely modulation of carrier density in the junction and contact length (L_con_) scaling shows the potential for all-carbon based electronics.

**Electronic supplementary material:**

The online version of this article (10.1186/s40580-017-0130-1) contains supplementary material, which is available to authorized users.

## Introduction

One of the key issues in future electronic devices is making ultra-short channel length (L_ch_) transistors with high performance in order to increase the density of each device. Therefore, CNTs and graphene are perspective electrical materials for future devices due to their high mobility, high on/off ratio and flexibility, with the sizes down to < 10 nm [1–4]. Therefore, all-carbon thin-film transistors using CNT or graphene as electrodes and channels are expected to enable the fabrication of flexible, transparent and high performance devices using relatively simple and low-cost techniques [5]. However, the full-potential of those exceptional properties can only be fully realized if a transparent, ohmic contact with negligible contact resistance contact is formed between them.

Contact resistance is an inherent property contributing to the total resistance of a system, which can be attributed to the contacting interfaces of electrical leads and connections (as opposed to the intrinsic resistance). In case of metal-CNT and CNT–CNT contact devices, the existence of a Schottky barrier at the contact area induces high contact resistance, limiting important performance parameters such as on-state current and field effect mobility. To improve the performance of CNT devices, a good electrical contact must be developed. As a member of the carbon allotropes, Graphene has emerged as an alternative electrode material for CNT devices due to its flexibility, exceptional electric transport, and potential for the seamless contact with CNT. While there have been several experimental and theoretical studies on the graphene/SWCNT junction properties, relatively few studies have been reported values of contact resistance in graphene/SWCNT junctions [6, 7].

Here, we define electrical contact resistance between graphene and SWCNT junction using the transfer length method (TLM) with a simple equivalent model. We have fabricated aligned arrays of SWCNTs with graphene electrode channel TFTs on Si/SiO_2_ substrates, with various L_ch_s (4–10 μm) and L_con_s (5–25 μm). The electrical transport properties indicate that p–n like junctions are formed in graphene/SWCNT junctions and exhibit contact resistances of ~ 494 and ~ 617 kΩ in case of each m-SWCNT and s-SWCNT, respectively. In addition, we find that contact resistance depends significantly on the carrier density and L_con_ even in micrometer regime.

## Experiment

### Synthesis of graphene on Cu foil

Single layer graphene was grown on the prepared 25 μm thick Cu foil (Alfa Aesar, 13,382) using chemical vapor deposition (CVD) method. The Cu foil was annealed in a quart tube at 1000 °C for 20 min under vacuum at < 10^−3^ torr with flowing H_2_ (10 sccm). Thereafter, a mixed gas of CH_4_ and H_2_ was injected for 20 min, and the Cu foil is rapidly cooled down to room temperature. Then the graphene was transferred on the Si/SiO_2_ wafer using PMMA as a supporting polymer layer in order to develop TFTs with graphene electrodes [8]. To fabricate two isolated electrodes (source and drain), the lithography process was performed with O_2_ plasma treatment.

### Synthesis of SWCNTs on quartz substrate

Aligned SWCNTs were grown on the pre-annealed (10 h, 1000 °C in air) ST-cut quartz (cutting angle: 42° 45′, Hoffman Materials, USA) substrate using CVD method. First, catalyst deposited quartz substrate (5 Å, deposited by e-beam evaporator) was inserted into a quartz tube and then heated up to 920 °C under ambient pressure with flowing Ar gas (1000 sccm) for pre-annealing of 1 h. After the pre-annealing, mixture of Ar (50 sccm), H2 (10 sccm) and CH4 (150 sccm) gas was injected to reaction chamber for 20 min for SWCNTs growth. The quartz substrate was rapidly cooled down to room temperature under Ar flow (1000 sccm) followed by the SWCNT growth [9]. As-grown SWCNTs were transferred onto patterned graphene/SiO_2_/Si substrate by using PMMA [Poly (methyl methacrylate)] as a supporting layer, similar to the graphene transfer method.

### Fabrication of graphene/SWCNT devices

A mask aligner system (MIDAS system Co., Ltd.) was used for the patterning of graphene, SWCNTs, and metal pad lines on the Si/SiO_2_ substrate in which silicon and silicon dioxide served as back-gate electrode and dielectric, respectively. To isolate each sets of TLM test structures, the pattern was formed by depositing a positive photoresist (AZ5214) and then etching by oxygen plasma. After removing the photoresist with acetone, the samples were annealed in a furnace under vacuum (10^−4^ torr) with Ar flow (500 sccm) at 400 °C for 3 h to remove PMMA and photoresist residue. The Cr/Au (~ 3–50 nm) pad electrode was e-beam evaporated on the top of graphene electrode in a vacuum with a pressure of 10^−7^ torr.

### Characterization of graphene/SWCNTs TFTs

The microstructure of the graphene/SWCNT TFTs were observed with a scanning electron microscope (SEM) (Hitachi S4800) and atomic force microscopy (AFM). We analyzed the electrical characteristics using a simple equivalent circuit model in which we assume diffusive transport in the channel. The quality of graphene/SWCNT TFTs were characterized by Raman spectroscopy (SENTERRA, Bruker GmbH, 532 nm). All electrical properties were measured by a probe station (Keithley 4200-SCS).

## Results and discussion

**Fig. 1:**
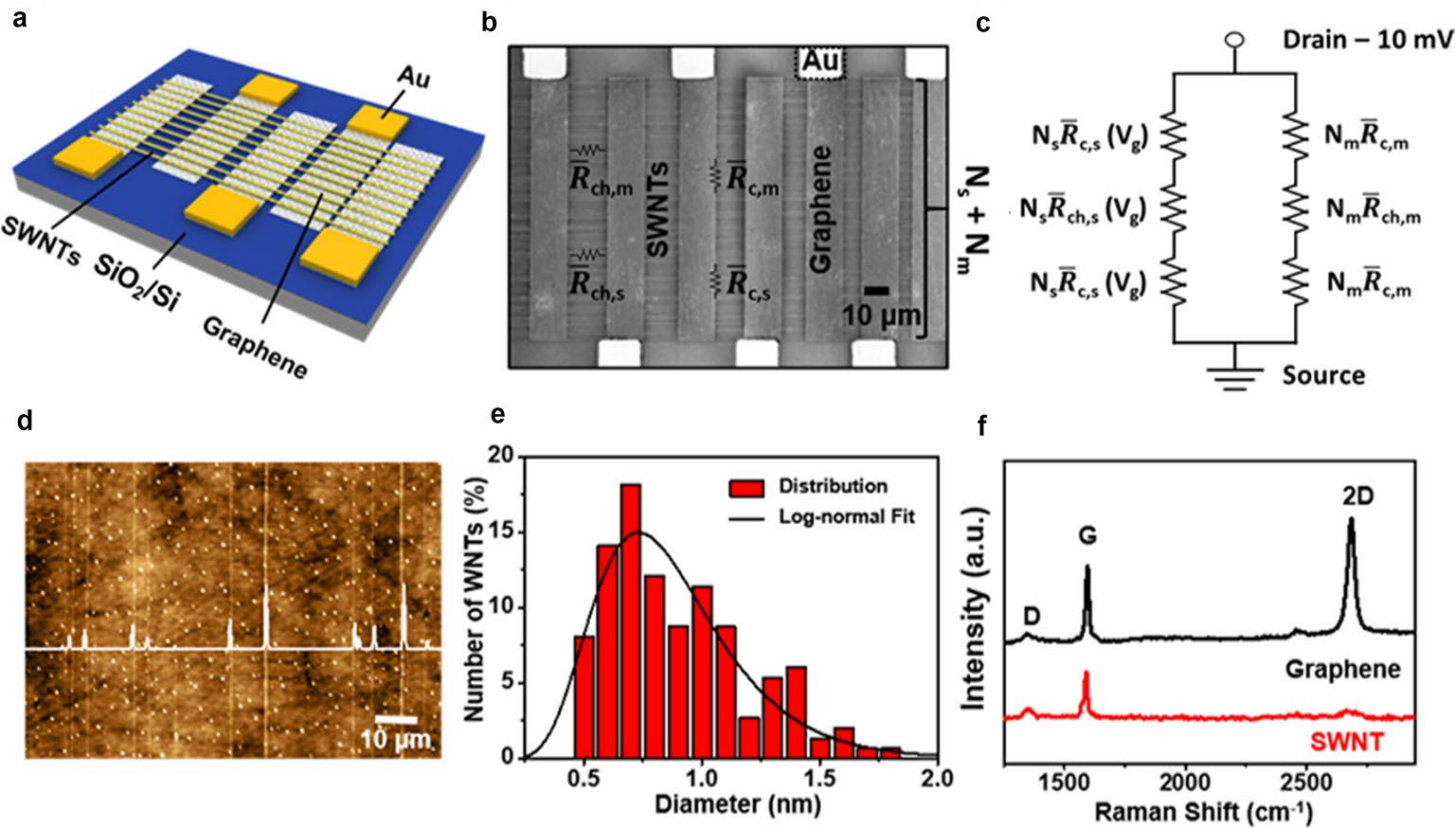
Schematic illustration (**a**) and SEM image (**b**) of graphene/SWCNTs junction TLM test structure. **c** Equivalent circuit model for graphene/SWCNTs junction transistor. **d** AFM image of CVD grown SWCNTs. **e** Typical diameter distribution of SWCNTs measured by AFM. **f** The Raman spectra for graphene and SWCNT

Figure 1a shows a fabricated graphene/SWCNTs junction TFT on Si/SiO_2_ (100 nm) substrate. The single layer graphene was synthesized on the Cu foil with a conventional CVD method, as reported in our previous work [9]. The highly aligned SWCNTs were fabricated from a CVD growth process with patterned metal catalyst on quartz substrate. They were then transferred sequentially onto Si/SiO_2_ substrates by using PMMA as a supporting layer [8]. Detailed description for the fabrication process is given in Sect. 2. During the CVD growth process, aligned SWCNTs are grown with 300 μm length between the patterned catalyst lines. The device layout consists of three separately fabricated parts: the SWCNTs channel, the graphene electrodes and the Au probing pads. The L_ch_ of the devices was defined by various spacing of patterned graphene (2, 4, 6, 8, 10, 12 and 14 μm) and overlapping length and width (25 and 100 μm, respectively) with aligned SWCNTs. The additional geometric information of graphene/SWCNTs device was shown in Additional file 1: Figure S1. To prevent damage of graphene electrodes while electrical performance was measured, the Au probing pads (~ 50 nm) were deposited by e-beam evaporator on the top of the graphene electrodes. Figure 1b shows an exhibits SEM image of representative L_ch_s of aligned SWCNTs with the graphene electrodes and probing pads at each device. In previous reports, it was determined that the diameter of individual SWCNTs is an important factor in the device behavior, a result attributed to the band gap parameter of the SWCNTs [10]. Moreover, it provides the Schottky barriers at the contact region between channel and electrode due to the different energy band level. To separate the influence of resistance induced in contact region, the diameter distribution of aligned SWCNTs on a SiO_2_ substrate was measured by AFM as shown in Fig. 1d, e. The densities of SWCNTs were 0.8 ± 0.2 SWCNTs μm^−1^ and average diameter distribution at ~ 0.87 nm. Moreover, for the differentiation of the SWCNTs chirality in this sample, AFM images were used with Raman spectra of SWCNTs to measure the diameter distribution. Consequently, we assume that the ratio of semiconducting SWCNTs to metallic SWCNTs is 63:37, and both the SWCNTs (channel) and graphene (electrode) were observed to have few defects in Fig. 1f. According to the chirality ratio of SWCNTs, we analyzed the characteristics using a simple equivalent circuit model, as shown in Fig. 1c. The main parameters for the model, which was used to estimate the contact resistance of between graphene/metallic SWCNTs and graphene/semiconducting SWCNTs respectively, are N_m_, N_s_ (the number of metallic and semiconducting SWCNTs, respectively), $$\begin{aligned} \hfill \\ {\bar{R}}_{{\text{c},\;\text{m}}} ,\,{\bar{R}}_{{\text{c},\;\text{s}}} \hfill \\ \end{aligned}$$(the average contact resistance of metallic and semiconducting SWCNTs between graphene, respectively), $$\begin{aligned} \hfill \\ {\bar{R}}_{{\text{ch}, \text{m}}} \hfill \\ \end{aligned}$$ and $$\begin{aligned} \hfill \\ {\bar{R}}_{{\text{ch},\;\text{s}}} \hfill \\ \end{aligned}$$ (the channel resistance of individual metallic and semiconducting SWCNTs, respectively). The control of the semiconducting tubes by the gate voltage is characterized using 10 mV current located between the source and drain.

**Fig. 2:**
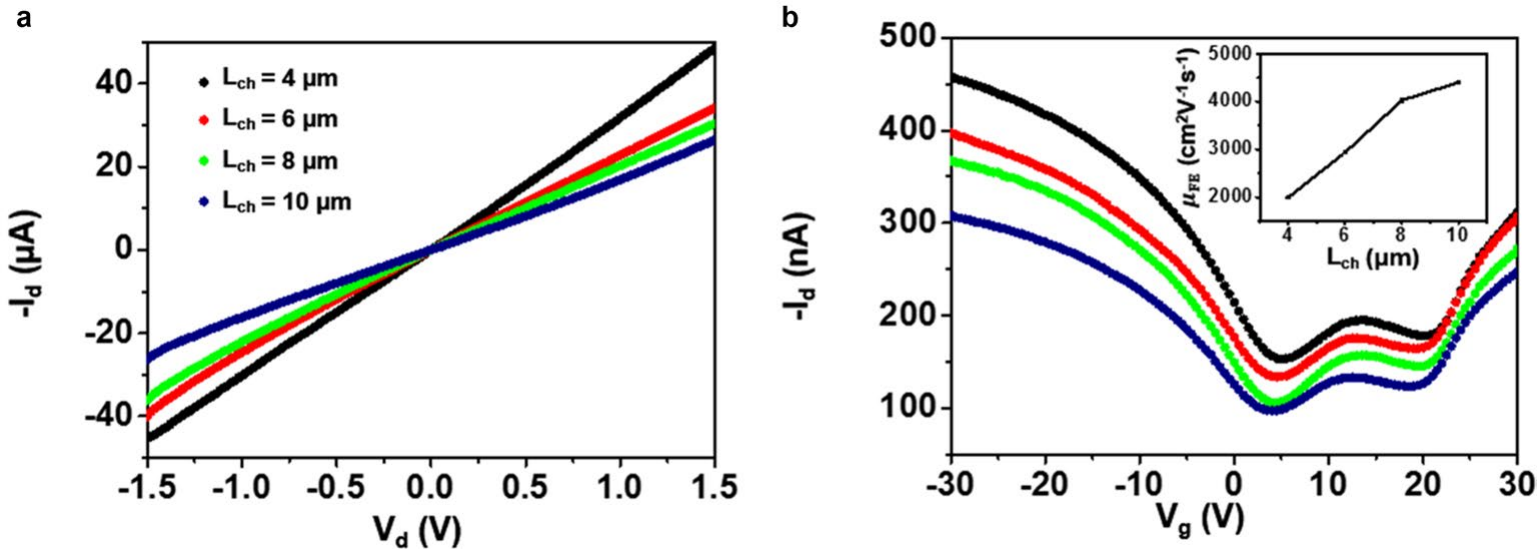
**a** I_d_–V_d_ characteristics (**b**) I_d_–V_d_ transfer characteristics of graphene-SWCNT TFT with different L_ch_ and a 100 μm width

Figure 2a shows the I_d_–V_d_ output characteristics of the graphene/SWCNTs transistors with typical L_ch_ (4, 6, 8 and 10 μm). The L_con_ and channel width were 25 and 100 μm, respectively. The overall drain current responses linearly with drain biases, indicating an ohmic-like contact behavior of graphene/SWCNTs junctions. From the transfer characteristics of graphene-SWCNTs transistors, measured with the source grounded, the drain voltage held at − 0.01 V, and gate voltage swept between ± 30 V (Fig. 2b), all curves exhibit ambipolar characteristics with two separated peaks. This outcome can be explained by relative p–n junction in the graphene/SWCNTs contact caused by the work function difference at the contact area [11]. Also, the transfer curve clearly shows the p-type doing of graphene and SWCNTs induced by the external adsorbates such as O_2_, CO_2_ and H_2_O. The on/off ratio ranged from ~ 2.96 to ~ 3.46, which comparable result to previous results on the metal electrode/SWCNT TFTs [3]. The inset of Fig. 2b shows calculated mobilities from devices with different L_ch_s. From the transfer curves, field effect mobilities can be calculated using the mobility calculation method. The values are as high as from 1991 to 4407 cm^2^V^−1^s^−1^. However, the monotonic decrease in mobility with decreasing L_ch_s suggests that the contact resistance plays a non-negligible role in the device operation [12, 13].

**Fig. 3:**
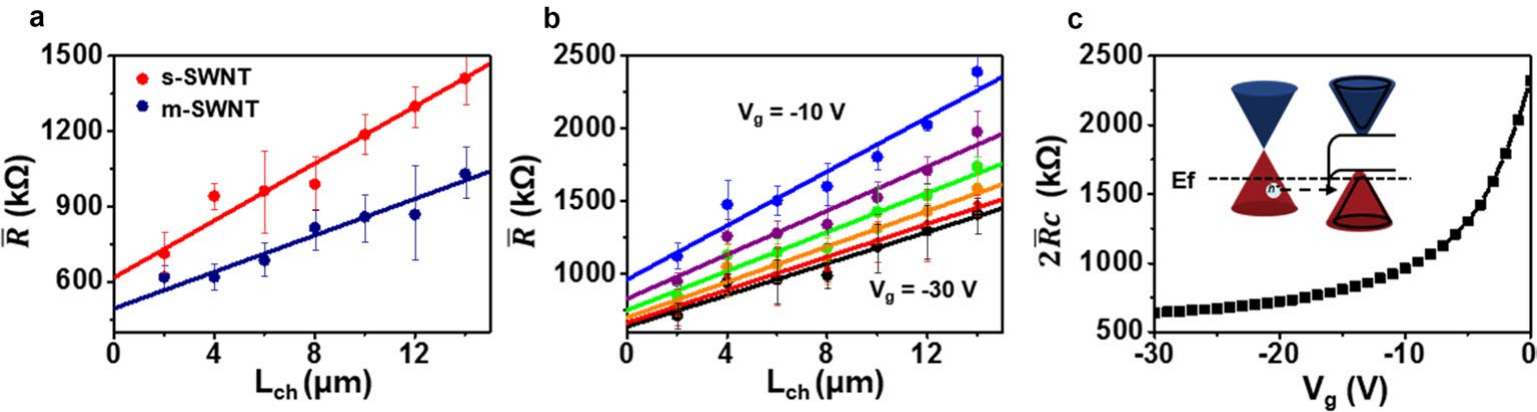
L_ch_ scaling and extracted contact resistance. **a** The average resistance of the m-SWCNTs and s-SWCNT as a function of L_ch_ at gate voltages of 0 V (m-SWCNT) and − 30 V (s-SWCNT), respectively. **b** The average resistance of the s-SWCNT as a function of L_ch_, varying the back-gate from − 30 to − 10 V with steps of 4 V. **c** The extracted contact resistance of the s-SWCNTs as a function of gate voltage

According to electrical device characteristics of graphene/SWCNT TFTs as shown in Fig. 2, the contact resistance for m-SWCNT and s-SWCNT are extracted from the TLM within diffusive transport regime (Fig. 3a). The 2-point total resistance $\text{R}_{{\text{tot}}}$ can be approximately written as the equations below:

$${\text{R}}_{{\text{tot}}} = {\rho}_{\text{SWNT}} {\text{L}}_{\text{ch}} + 2{\text{R}}_{\text{c}}$$

where $\rho_{{\text{SWNT}}}$ is the resistivity of SWCNTs and R_c_ is the contact resistance between graphene and SWCNTs. The slope of the plot gives the resistivity of SWCNTs. The y-intercept of the total resistance as a function of L_ch_ indicates the contact resistance. Since the m-SWCNTs are, nominally, not modulated by the gate bias, we consider only s-SWCNTs in an approximate procedure that involves subtracting the minimum current which occurs at V_g_ near 5 V for devices reported here, from the measured currents used in the TLM test structure. Therefore, the resistivity of SWCNTs was determined to be around 36 kΩ μm^−1^ (m-SWCNT) and 56 kΩ μm^−1^ (s-SWCNT), respectively. Also, the contact resistance between graphene and SWCNTs at fixed contact area (25 by 100 μm) was estimated to be around 494 kΩ (m-SWCNT) and 617 kΩ (s-SWCNT), respectively. As the V_g_ in the channel is increased from − 30 to − 10 V with steps of 4 V, the contact resistance increases from 617 to 2316 kΩ (Fig. 3b). This means that the decrease of contact resistance at lower bias is related to the increase of electrostatically doped carrier density in both graphene and SWCNTs. The higher carrier density in the junction induced by negative back-gate bias enhances the carrier concentration, which leads to a narrower Schottky barrier (Fig. 3c). Meanwhile, the narrowed Schottky barrier would thus facilitate the hole injection from the graphene into the SWCNTs, leading to a lower contact resistance.

**Fig. 4:**
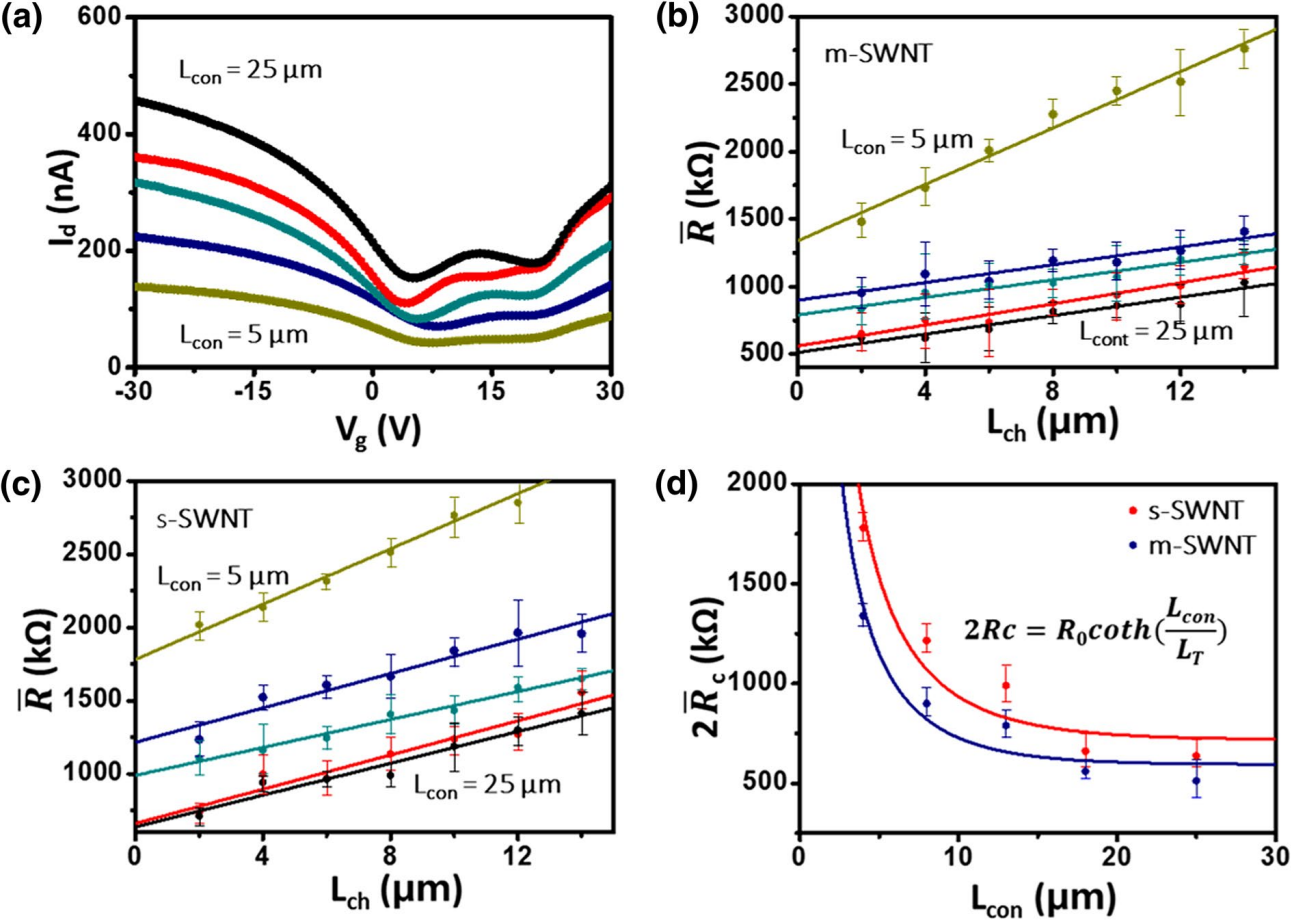
**a** I_d_–V_g_ transfer characteristics of graphene-SWCNT TFT with different L_con_ (5, 10, 15, 20, 25, 30 μm) and a 10 μm channel width. The average resistance of the m-SWCNTs (**b**) and s-SWCNT (**c**) as a function of L_ch_ at different L_con_. **d** The extracted contact resistance as a function of L_con_ and fitting line

L_con_ scaling was studied in the same manner as L_ch_ scaling. Figure 4a shows a plot of transfer curves of different L_con_ (5, 10, 15, 20, and 25 μm) graphene/SWCNT junction TFTs. Figure 4b, c shows average resistance of m-SWCNT and s-SWCNT with different L_con_s. In the same manner as above, contact resistances were extracted and plotted on Fig. 4d [14]. The results indicate that as L_con_ shortened, the resistance increased. Some studied have suggested that transport between a metal and a SWCNT occurs only at the contact edge, with no L_con_ dependence [15–17]. This differing tendency is caused by longer transfer length.

$$\text{L}_{\text{T}} = \frac{{2\text{R}_{\text{c}} }}{{\rho_{{\text{ch}}} }}$$

The transfer length (L_T_), which is the length along a contact at which the applied voltage drops to 1/e of its value, is extrapolated from line intercepts from the x-axis of the TLM plot. The best fit to the data using equation below is shown by solid curve. The determined L_T_ from fitted line are 8.74 and 9.75 μm for m-SWCNT and s-SWCNT, respectively. The experimental data agree with the fitting model. This suggest that the contact resistance depends significantly on L_con_ even in micrometer regime due to the long transfer length.

## Conclusions

In conclusion, we have explored the contact resistance in graphene/SWCNT junctions using TLM with a simple equivalent model. The Schottky barrier between graphene and SWCNT leads to a p–n like junction. We investigated the contact resistance modulation of graphene/SWCNT junctions, which is controlled by back-gate bias. From our measurement, the contact resistance in the junction was found to be ~ 494 and ~ 617 kΩ in the case of m-SWCNT and s-SWCNT. We confirmed that the work function tuning of graphene and SWCNT induced by doping and controlling the L_con_ can alter the contact resistance in graphene/SWCNT junctions. Moreover, further development of contact engineering such as investigation of SWCNT chirality and lowering the defect density of graphene shows the potential for all-carbon based electronics.

## Additional file

**Additional file 1: Figure S1.** Additional geometric information of graphene/SWCNT devices.

## Abbreviations

SWCNT: single-walled carbon nanotube
M: metallic
S: semiconducting
L_con_: contact length
L_ch_: channel length
TLM: transfer length method
CVD: chemical vapor deposition
SEM: scanning electron microscope
AFM: atomic force microscopy
PMMA: Poly (methyl methacrylate)
N_m_, N_s_: the number of metallic and semiconducting SWCNTs, respectively
${\bar{R}}_{{\text{c}, \text{m}}} ,{\bar{R}}_{{\text{c},\text{s}}}$: the average contact resistance of metallic and semiconducting SWCNTs between graphene, respectively
$$\begin{aligned} \hfill \\ {\bar{R}}_{{\text{ch,}\;\,\text{m}}} \hfill \\ \end{aligned}$$ and $$\begin{aligned} \hfill \\ {\bar{R}}_{{\text{ch,}\;\,\text{s}}} \hfill \\ \end{aligned}$$: the channel resistance of individual metallic and semiconducting SWCNTs, respectively
L_T_: transfer length
