# Supplementary material for: Analysis of contact resistance in single-walled carbon nanotube channel and graphene electrodes in a thin film transistor
Source: Nano Converg. 2017 Dec 20;4:35. doi: 10.1186/s40580-017-0130-1 (PMC5736787; doi:10.1186/s40580-017-0130-1)
Supplement: Supplementary file 1 — Additional file 1: Figure S1. Additional geometric information of graphene/SWCNT devices. [file 40580_2017_130_MOESM1_ESM.docx]

**Supporting Information**

Analysis of contact resistance in single-walled carbon nanotube channel and graphene electrodes in a thin film transistor

*Jinwook Baek*^†^*, Travis G. Novak*^†^*, Houngkyung Kim*^†^*, Jinsup Lee*^†^*, Byoungwook Jang*^†^*,*

*Junseok Lee*^†^*, and Seokwoo Jeon*^†,^*

^†^ Department of Materials Science and Engineering,
Korea Advanced Institute of Science and Technology,
Daejeon 305-701, Republic of Korea.

* Corresponding author. Tel: +82 42 350 3342. E-mail: [jeon39@kaist.ac.kr](mailto:jeon39@kaist.ac.kr)


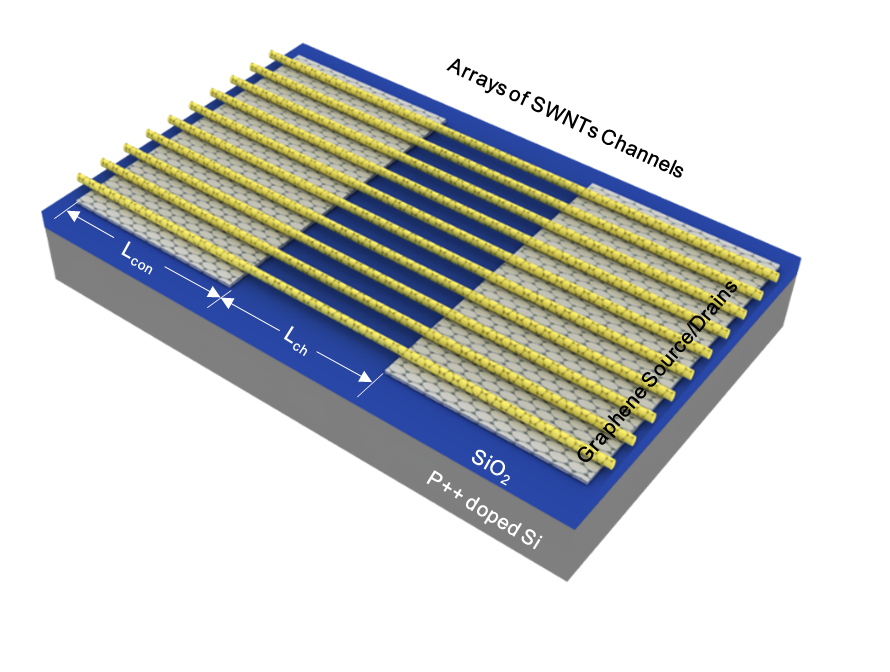


Figure S1. Additional geometric information of graphene/SWCNT devices.
